# Second Primary Malignancies in Gastrointestinal Cancer Survivors: Epidemiology, Risk Factors, and Future Prediction Models

**DOI:** 10.3390/cancers18193195

**Published:** 2026-10-03

**Authors:** Jyoti Yadav, Thomas Balizzakiwa, Hemant Goyal

**Affiliations:** 1 The Wright Center for Graduate Medical Education, Scranton, PA 18505, USA; yadavj@thewrightcenter.org (J.Y.); balizzakiwat@thewrightcenter.org (T.B.); 2 Borland Groover, Baptist Medical Center-Downtown, Jacksonville, FL 32207, USA

**Keywords:** second primary malignancy, gastrointestinal cancer, cancer survivorship, competing risk, Lynch syndrome, field cancerization, SEER, nomogram

## Abstract

Advances in gastrointestinal (GI) cancer care have produced a growing population of long-term survivors at risk of second primary malignancies (SPMs). SPM definitions vary across studies, and SPMs remain underrepresented in cancer forecasting and prediction tools for GI cancer survivors. We conducted a narrative review across major GI cancer subtypes, summarizing site-specific SPM risk, key etiological pathways, and available prediction tools. Approximately one in ten colorectal cancer (CRC) survivors develops an SPM at ten years (pooled SIR ~1.15), a figure specific to CRC that does not generalize to other GI sites. Gastric cancer survivors carry excess risk of thyroid (O/E 2.00), esophageal, small intestinal, and pancreatic (O/E 1.60) malignancy, amplified by radiotherapy. Esophageal squamous cell carcinoma survivors show markedly elevated risk of upper aerodigestive tract tumors, consistent with field cancerization. Lynch syndrome (~16% second CRC risk at 10 years) and familial adenomatous polyposis (near-100% lifetime CRC risk without colectomy) confer some of the highest lifetime risks of metachronous GI malignancy reported to date. Most forecasting frameworks treat cancer incidence as a single event and do not adequately capture survivor-specific transitions, competing mortality, or germline susceptibility. GI cancer survivors form a distinct group whose risk is often overlooked. Harmonizing SPM definitions, extending multistate and competing-risk modeling, and integrating hereditary-risk stratification are near-term priorities for individualized surveillance.

## Simple Summary

People who survive cancers of the digestive system, such as the colon, stomach, esophagus, liver, and pancreas, are living longer than ever. Longer survival also means more time in which a new, unrelated cancer can develop. These second cancers are an important cause of illness and death among survivors, yet different studies count them in different ways, and they are rarely built into the tools doctors use to estimate future risk. Here, we bring together what is known about how often second cancers occur after each type of digestive cancer, which organs are most often affected, and why. We describe four main drivers: shared habits such as smoking and drinking, side effects of earlier treatment, widespread damage to the lining of the digestive tract, and inherited cancer syndromes. We then assess current risk-prediction tools and outline practical steps that could help doctors tailor follow-up care to each survivor’s level of risk.

## 1. Introduction

Improvements in early detection, systemic therapy, and multimodal treatment have substantially extended survival after a diagnosis of gastrointestinal (GI) cancer. An estimated 18.6 million people in the United States are now living with a history of cancer, and this number is projected to exceed 22 million by 2035 as screening and treatment continue to improve [1]. One consequence of this success is a longer survivorship window: survivors now live long enough to develop a second, biologically distinct malignancy, a second primary malignancy (SPM), rather than a recurrence or metastasis of their original tumor.

The study of SPMs is complicated at its foundation by inconsistent case definitions. The Surveillance, Epidemiology, and End Results (SEER) program applies latency rules and complex, site-specific coding logic to distinguish a genuine new primary from recurrent or metastatic disease. Many single-institution and multinational cohort studies instead apply a uniform six-month interval to separate “synchronous” from “metachronous” second cancers [2,3,4]. International Association of Cancer Registries/International Agency for Research on Cancer (IACR/IARC) rules add a further layer of topography and histology-specific criteria. Because these frameworks classify borderline cases differently, reported SPM incidence for the same clinical scenario can vary appreciably between registry-based and cohort-based studies. A model trained on one definitional standard may simply not transfer to a population characterized under another.

Cancer survivors are increasingly recognized as a distinct clinical population, yet population-level forecasting models and individualized prediction tools have been slow to incorporate SPM risk in any systematic way. GI cancer survivors are especially underserved in this respect. This is a heterogeneous group spanning esophageal through colorectal primaries including the liver and pancreas. It lags breast, prostate, and hematologic malignancy survivors, for whom more mature risk-prediction tools already exist [5,6]. This narrative review has three aims. The first is to synthesize the epidemiology of SPMs across major GI cancer subtypes. The second is to examine the mechanistic and clinical factors that drive this excess risk. The third is to critically evaluate the current state of forecasting and prediction modeling, with the goal of identifying concrete priorities for survivorship-focused research.

## 2. Methods

We conducted a structured narrative review, reflecting the breadth of the topic across distinct GI cancer subtypes and methodological traditions (registry epidemiology, hereditary cancer genetics, radiation oncology, and biostatistical modeling). We searched PubMed and supplementary sources for SEER-based registry studies, multi-institutional cohort analyses, hereditary cancer syndrome databases and guideline statements, and the predictive-modeling literature published predominantly within the last two decades, reflecting the period during which modern multimodal GI cancer therapy has meaningfully extended survivorship and during which most SEER-based SPM epidemiology and competing-risk modeling methodology has been developed, focusing on survivors of colorectal, gastric, esophageal, hepatocellular, pancreatic, and small-bowel cancers. Search terms combined “second primary malignancy,” “second primary cancer,” “multiple primary cancer,” and “metachronous”/”synchronous” tumor with individual GI cancer subtypes, relevant hereditary syndromes (Lynch syndrome, familial adenomatous polyposis), and modeling terms (competing risk, nomogram, multistate model, standardized incidence ratio). We prioritized large registry-based cohorts (SEER, national cancer registries), meta-analyses, and studies that explicitly reported standardized incidence ratios (SIRs), excess absolute risk (EAR), or validated prediction models. We extracted and compared definitions of SPM latency across included studies, characterized major risk factors (shared lifestyle exposures, treatment-related carcinogenesis, hereditary predisposition, field cancerization), and critically evaluated existing forecasting and individualized risk-prediction frameworks for their incorporation of survivor-specific parameters.

Because this is a narrative review, no protocol was pre-registered, study selection was not conducted by independent dual reviewers, and no formal risk-of-bias or quality appraisal instrument was applied to included studies; consequently, the synthesis below should be read as a structured overview of the existing evidence rather than a pooled or meta-analytic estimate. Numeric findings are reported as they appear in the source study and are explicitly attributed to the specific GI cancer subtype, cohort, and follow-up duration from which they were derived; unless a finding is explicitly described as pooled or meta-analytic (e.g., the colorectal cancer standardized incidence ratio meta-analysis discussed in Section 3.2), estimates for one GI cancer subtype should not be extrapolated to other subtypes or to GI cancers as a group.

## 3. Results

### 3.1. Definitional Heterogeneity in SPM Ascertainment

Before comparing risk estimates across studies, it is important to recognize that “SPM” is not a uniformly defined construct. SEER’s multiple-primary rules classify tumors by topography, histology, and laterality with a nominal latency threshold, generating conservative estimates that exclude many early second cancers as manifestations of the index tumor. Cohort studies more commonly define synchronous SPMs as those detected within six months of the index diagnosis and metachronous SPMs as those detected thereafter [2,3]. IACR/IARC criteria incorporate additional histology-grouping rules that can further split or lump specific tumor pairs. Registry-based reviews of SPM after cancer note that reported prevalence ranges from roughly 6.6% to 9% depending on registry and population, with a two- to six-fold increase in relative risk in specific high-risk subgroups such as childhood cancer survivors [4]. This heterogeneity has direct consequences for survivorship research: pooled or comparative analyses that do not harmonize latency definitions risk conflating true biological differences in SPM risk with artifacts of case ascertainment. In our view, this is one of the more fixable weaknesses in the field. Unlike biological heterogeneity across GI cancer subtypes, definitional heterogeneity is a matter of reporting convention, and could in principle be resolved by requiring studies to report results under more than one latency framework. To improve comparability, future registry and cohort studies could adopt a dual-reporting approach, presenting a primary analysis under the registry’s own multiple-primary rules (SEER or IACR/IARC [7]) alongside a sensitivity analysis using a uniform 6-month synchronous/metachronous cutoff, and stating how many second primaries are reclassified between the two definitions. Where standardized incidence ratios are reported, presenting them with and without the 2-month latency exclusion conventionally applied in SEER-based analyses [8], and stratified by time since the index diagnosis, would help distinguish early excess risk, which largely reflects detection during staging and initial follow-up, from later excess risk that is more likely to reflect shared etiology or treatment effects. Clearly stating the definitional framework in the abstract would also facilitate future pooled analyses. Table 1 summarizes the three definitional frameworks referenced throughout this review.

### 3.2. Colorectal Cancer Survivors

Colorectal cancer (CRC) survivors are the most extensively studied GI cancer population with respect to SPM risk. In a large SEER analysis of 179,884 CRC patients aged 20–79 years followed for up to 18 years, the largest single-cohort estimate of absolute SPM risk in CRC survivors to date; 10.2% developed an SPM, with 3, 5, and 10-year cumulative risks of 3.9%, 5.9%, and 10.0%, respectively, and a median survival after the second diagnosis of under 4 years [9]. A separate SEER-based study of 140,907 CRC survivors developed sex-specific risk models after finding that 10-year cumulative incidence of second primary cancer was higher in men than women; the most common second cancers were breast, lung and bronchus, and pancreatic cancers in women, and prostate, lung and bronchus, and urinary tract cancers in men: findings the authors used to construct separate predictive nomograms for female and male CRC survivors [10]. A pooled meta-analysis of 42 studies (SIR 1.15 with no lag restriction) found the most consistent excess risk for second primary cancers of the colorectum, corpus uteri (endometrium), and small intestine among CRC survivors; evidence for prostate, breast, and several other sites was reported but judged more limited and less consistent across studies [11]. One caveat is worth stating plainly: the 10.2% ten-year cumulative risk in [9] and the pooled SIR of 1.15 in [11] answer different questions: absolute risk within a single cohort versus relative risk against the general population across heterogeneous cohorts. The two should not be added or compared directly. These CRC-specific figures should not be extrapolated to other GI cancer subtypes, which show materially different SPM incidence, site patterns, and follow-up horizons, as detailed below.

Age at diagnosis meaningfully modifies this risk. Young-onset CRC survivors (diagnosed before age 50) show a 44% excess relative risk of SPM compared with a near-null excess in older survivors, with markedly elevated site-specific SIRs for small intestinal cancer in this younger group, a pattern consistent with an underlying hereditary or field-level susceptibility in early-onset disease [12]. CRC survivors are also at recognized excess risk of a second primary pancreatic cancer, a relationship first characterized in an Asian cancer-registry cohort and subsequently corroborated in Western populations, with implications for pancreatic surveillance strategies in long-term CRC survivors [13]. Notably, tumor sidedness also appears to influence the pattern of subsequent GI primaries: in a SEER cohort of 281,413 CRC patients, 4.3% developed a second primary GI malignancy over a median 4.9-year follow-up, with right-sided colon cancer carrying a significantly higher standardized incidence ratio for second primary GI malignancy than left-sided colon or rectal cancer [14]. Whether tumor sidedness and early age at onset interact to modify metachronous GI risk has not, to our knowledge, been formally examined. The SEER sidedness analysis did not report age-stratified estimates [14]. In a cohort comparing early- and late-onset CRC, right-sided primaries carried the highest SPM risk in multivariable analysis (hazard ratio 1.70 compared with rectal primaries), but sidedness-specific estimates within the early-onset group and an age-by-site interaction term were not reported [15]. A meta-analysis of 7865 patients with early-onset CRC found a pooled metachronous CRC incidence of 2.6%, comparable to that of older patients, with the excess concentrated among patients with Lynch syndrome (18.4%), microsatellite instability (19.7%), or a family history of CRC (10.5%) [16]. Because right-sided location and early onset are both enriched for mismatch-repair deficiency, their apparent effects may partly reflect a shared hereditary substrate rather than independent or synergistic risk. Registry analyses incorporating an explicit age-by-sidedness interaction term, ideally adjusted for mismatch-repair or microsatellite-instability status, are needed to address this question.

### 3.3. Gastric Cancer Survivors

Gastric cancer survivors demonstrate a distinct SPM risk profile. A US population-based SEER study found significantly increased observed-to-expected ratios for second gastrointestinal malignancies (O/E 1.71), thyroid cancer (O/E 2.00), and pancreatic cancer (O/E 1.60), with a median time to first SPM of nearly four years; risk of second melanoma, breast, and prostate cancer were, by contrast, lower than expected [17]. Radiation exposure further modified site-specific risk: among irradiated gastric cancer survivors, the O/E ratios for thyroid, esophageal, and pancreatic SPMs were higher than among those without radiation exposure, while stomach and small-intestinal SPM risk remained elevated regardless of radiotherapy status [17]. Rarer gastric neoplasms follow a similar pattern. Survivors of gastric neuroendocrine tumors carry an overall SIR for any SPM of 2.09, with markedly elevated site-specific risk in the stomach, small intestine, thyroid, hepatobiliary tract, and pancreas [18]. Thyroid cancer specifically emerges as a recurring second-site malignancy across several GI cancer subtypes; a SEER latency-trend analysis found a 90% excess relative risk of second primary thyroid cancer across all prior cancer types combined, with elevated risk persisting for up to ten years after diagnoses of gastric, colonic, and pancreatic cancer, supporting incorporation of thyroid surveillance into long-term GI cancer survivorship planning [19].

### 3.4. Esophageal Cancer and Field Cancerization

Survivors of esophageal squamous cell carcinoma (ESCC) illustrate the concept of field cancerization particularly clearly. The term “field cancerization” itself originates from Slaughter’s 1953 description of multifocal, independent carcinogenesis across a chronically exposed epithelial field, and it remains the dominant explanatory framework for these multifocal squamous tumors [20,21]. A nationwide Western cohort study found a markedly elevated risk of synchronous and metachronous second primary tumors of the upper aerodigestive tract among ESCC survivors [2]. The reciprocal relationship is equally well established: in a Taiwanese cohort, 15–20% of patients with head and neck squamous cell carcinoma (HNSCC) developed a second esophageal primary, and vice versa, reflecting shared carcinogenic exposure, principally tobacco and alcohol, across the entire aerodigestive mucosa; this Asian-population figure is considerably higher than the pooled non-Asian prevalence described below, consistent with the well-documented geographic gradient in esophageal squamous cell carcinoma incidence [22]. A systematic review and meta-analysis pooling 26 studies (8071 patients) found an overall pooled prevalence of 5.4% for second primary tumors of the aerodigestive tract in non-Asian HNSCC populations (5.3% for esophageal second primary tumors specifically), notably lower than the up-to-17% figures drawn from earlier, smaller studies that motivated the review and nonetheless recommended endoscopic screening for esophageal second primary tumors, particularly in patients with an index hypopharynx carcinoma [20]. Prospective endoscopic surveillance cohorts using Lugol chromoendoscopy have shown that image-enhanced screening can detect metachronous esophageal neoplasia at earlier, more treatable stages, with an incidence of metachronous esophageal squamous neoplasia of roughly 11% over ten years of follow-up in one prospective series [23,24]. Notably, this field-cancerization relationship appears attenuated in human papillomavirus (HPV)-associated oropharyngeal cancer, where traditional concepts of a shared carcinogenic field are less applicable, underscoring that SPM risk stratification must account for tumor biology rather than anatomic site alone [25].

### 3.5. Hepatocellular, Pancreatic, and Other GI Malignancies

Hepatocellular carcinoma (HCC) survivors, an increasingly large population given advances in surveillance and locoregional therapy, show an overall SPM incidence of 3.95% in a SEER cohort of over 40,000 patients diagnosed between 2000 and 2014, with 3-, 5-, and 10-year cumulative incidences of 2.35%, 3.12%, and 4.51%, respectively; site-specific excess risk was significant for cancers of the oral cavity and pharynx, stomach, biliary tract, lung, kidney, thyroid, and lymphatic system [26]. Age at initial diagnosis, extent of disease, tumor size, and treatment were independent risk factors incorporated into a risk-prediction nomogram, which achieved a concordance index of 0.677 [26]. At first glance it seems paradoxical that favorable prognostic features, such as smaller tumors, earlier stage, and receipt of surgery, tracked with higher SPM incidence. We read this the same way the original authors did: it is a survivorship-window effect rather than a causal one. Only patients who live long enough are around to develop a second cancer at all, which is exactly why competing-risk methods, rather than standard survival analysis, are the right statistical tool here. Pancreatic and gastric carcinomas, in contrast, show comparatively low SPM detection (under 5% at 15 years), largely because their poor baseline prognosis limits the survivorship window during which a second cancer can emerge [4].

Because the magnitude, timing, and site pattern of SPM risk differ substantially across GI cancer subtypes, Table 2 consolidates the site-specific findings described above alongside the hereditary syndromes discussed in Section 3.7, to aid at-a-glance comparison; the individual citations in this table point to the specific study each estimate was drawn from, and none of these figures should be generalized beyond the index cancer or syndrome listed.

### 3.6. Treatment-Related Carcinogenesis

Radiotherapy is among the best-characterized modifiable contributors to SPM risk in GI cancer survivors. A SEER cohort study estimated that radiotherapy is causally attributable to a measurable proportion of second cancers among irradiated survivors overall, with risk that rises after a latency of approximately five years and continues to increase with time since exposure [32]. In rectal cancer specifically, pelvic external beam radiotherapy is associated with a significantly increased risk of second primary rectal cancer (SIR 1.22) and combined external beam plus brachytherapy carries an even higher risk (SIR 1.85); survival after a radiation-associated second rectal cancer is significantly worse than after a primary rectal cancer alone [33]. A related SEER analysis found that radiotherapy changes not only the magnitude but the pattern of second malignancy after rectal cancer, altering the distribution of subsequent primary sites compared with non-irradiated survivors, and noted the confounding possibility that some rectal cancer patients carry unrecognized Lynch syndrome, which independently elevates risk of second colorectal and gastric cancers regardless of treatment [34]. Modern radiotherapy techniques, including intensity-modulated radiotherapy, volumetric modulated arc therapy, and particle therapy, are designed in part to reduce integral dose to non-target tissue, but their long-term secondary malignancy profiles remain incompletely characterized because the requisite latency for solid tumor induction has not yet fully elapsed in patients treated with these newer modalities [35,36]. Dosimetric modeling nevertheless allows some informed expectations. The transition from conventional to three-dimensional conformal radiotherapy reduced the volume of normal tissue exposed to high doses, which would be expected to reduce in-field sarcomas. In contrast, IMRT and VMAT use more beam angles and two to three times as many monitor units, spreading low-dose exposure over a larger volume of normal tissue and increasing whole-body leakage dose; Hall and Wuu estimated that this could increase the incidence of radiation-associated second malignancies from approximately 1% to 1.75% among patients surviving 10 years [37]. For modern photon techniques, the predicted effect is therefore primarily a redistribution of second tumor sites rather than a uniform reduction in overall incidence, with fewer high-dose, in-field tumors but potentially more carcinomas in organs within the low-dose region; the net effect depends on organ-specific dose–response relationships that remain uncertain at higher doses [36,37]. Proton therapy, which reduces integral dose, is the modality most likely to lower overall incidence. For stereotactic ablative radiotherapy (SABR), the highly conformal treatment field reduces high-dose exposure to surrounding tissues, but long-term SPM risk remains uncertain because available cohorts have not yet reached sufficient latency for robust assessment, and current estimates derive largely from modeling studies [35]. Early clinical data are consistent with these predictions: in a National Cancer Database analysis of more than 450,000 patients followed for a median of 5.1 years, IMRT was associated with no overall difference in second cancer risk compared with three-dimensional conformal radiotherapy (adjusted odds ratio 1.00), whereas proton therapy was associated with a substantially lower risk (adjusted odds ratio 0.31), although short follow-up and potential residual confounding limit causal inference [38]. For GI cancer survivors, SPM site distributions derived from historical cohorts may therefore not apply directly to patients treated with contemporary techniques, and clinical attention may need to extend beyond the treated field to organs within the low-dose region.

### 3.7. Hereditary Cancer Syndromes

Hereditary predisposition syndromes account for a disproportionate share of clinically significant metachronous GI malignancy. Lynch syndrome, caused by germline mutations in the DNA mismatch repair genes MLH1, MSH2, MSH6, or PMS2 (or 3′ EPCAM deletions causing MSH2 silencing), confers a lifetime CRC risk of 50–70% and an endometrial cancer risk of 40–60%, alongside elevated lifetime risk (roughly 5–15%) of gastric, urinary tract, small intestinal, ovarian, biliary tract, brain, and sebaceous neoplasms [27,28]. Because mismatch-repair deficiency predisposes to ongoing carcinogenesis across the colorectal mucosa rather than a single focal event, Lynch syndrome carriers face an approximately 16% risk of a second metachronous colorectal cancer within ten years of an index diagnosis, a figure that directly informs decisions between segmental resection with intensive surveillance versus extended or total colectomy at the time of index surgery [28,39]. Pancreatic ductal adenocarcinoma risk in Lynch syndrome appears more modest than historically assumed: a recent nationwide Dutch cohort study found a combined lifetime risk of pancreatic, ampullary, and distal cholangiocarcinoma of only 0–3.4% across genotypes, below the 5% threshold conventionally used to justify pancreatic surveillance, suggesting that current surveillance recommendations in this specific context may warrant reappraisal [29].

Familial adenomatous polyposis (FAP), caused by germline APC mutations, carries a near-100% lifetime colorectal cancer risk in the absence of prophylactic colectomy, with adenomas present in approximately half of patients by age 15 and in 95% by age 35; carcinoma typically develops about a decade after polyp onset, so that most patients with an intact colon develop CRC by the age of 40 to 50 years [30,31]. Beyond the colorectum, FAP patients remain at long-term risk for desmoid tumors, duodenal and ampullary adenocarcinoma, and other extracolonic manifestations, reinforcing the need for lifelong, syndrome-specific surveillance protocols distinct from those used in sporadic CRC survivors [30]. Across both syndromes, genotype-specific and gene-specific risk stratification (for example, differential pancreatic risk by MLH1 versus MSH6 mutation status) is increasingly recognized as necessary to avoid both under- and over-surveillance [29].

**Figure 1:**
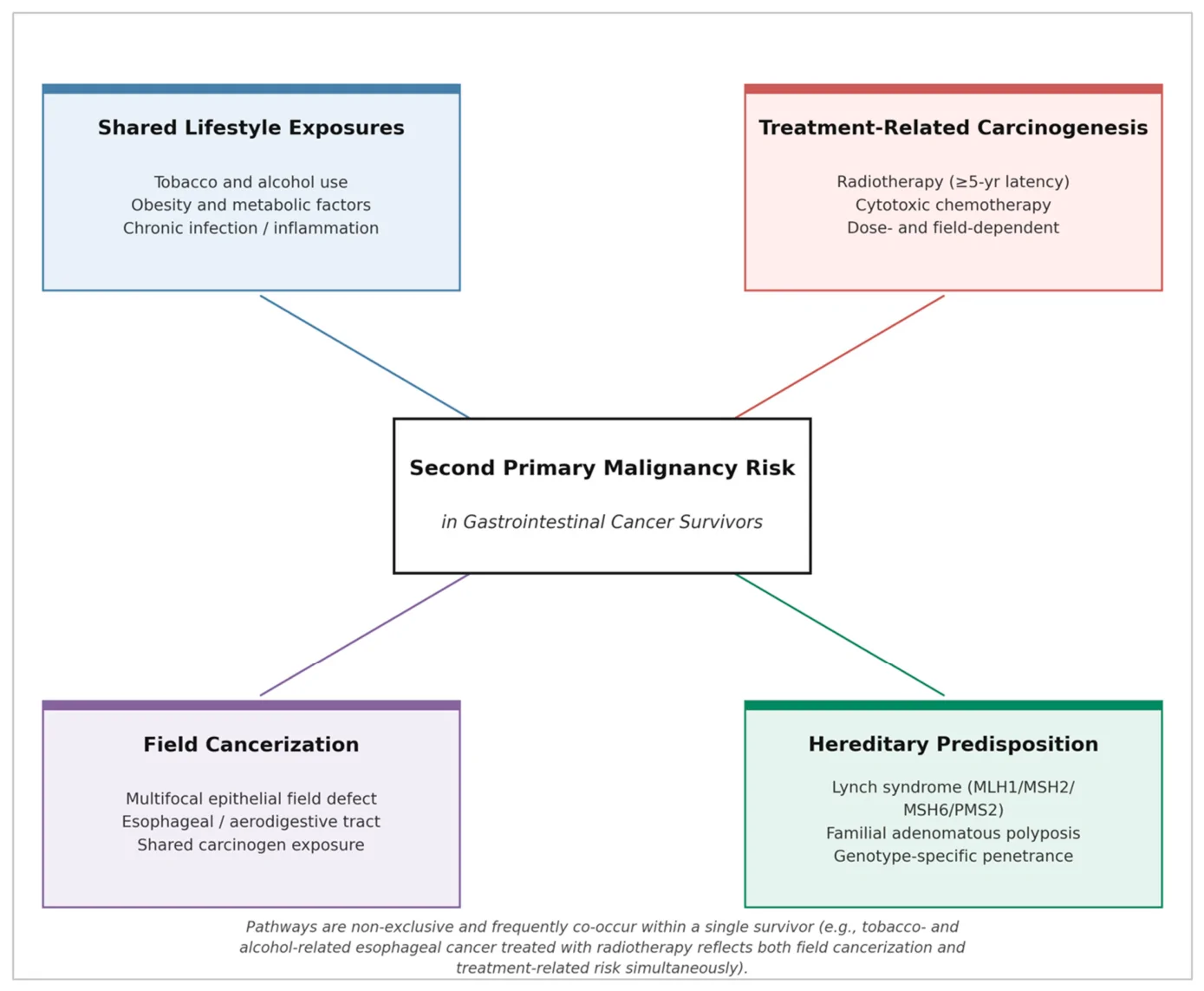
Conceptual framework of the four principal etiological pathways contributing to second primary malignancy (SPM) risk in gastrointestinal (GI) cancer survivors. Upper left (blue), shared lifestyle exposures: tobacco and alcohol use, obesity and metabolic factors, and chronic infection or inflammation, which affect multiple organs and underlie many of the site-specific associations described in Section 3.2, Section 3.3, Section 3.4 and Section 3.5. Upper right (red), treatment-related carcinogenesis: radiotherapy, with excess risk typically emerging after a latency of approximately 5 years and varying with dose and treatment field, and cytotoxic chemotherapy (Section 3.6). Lower left (purple), field cancerization: multifocal, independent carcinogenesis across a chronically exposed epithelial field, best illustrated by the reciprocal relationship between esophageal and head and neck squamous cell carcinoma (Section 3.4). Lower right (green), hereditary predisposition: germline syndromes including Lynch syndrome (MLH1, MSH2, MSH6, PMS2) and familial adenomatous polyposis, with genotype-specific penetrance (Section 3.7). Lines connecting each pathway to the central box indicate its contribution to overall SPM risk. The pathways are not mutually exclusive and frequently coexist in the same survivor (for example, tobacco- and alcohol-related esophageal cancer treated with radiotherapy); risk estimates derived for individual pathways should therefore not be summed.

Section 3.2, Section 3.3, Section 3.4, Section 3.5, Section 3.6 and Section 3.7 describe four overlapping, non-exclusive etiological pathways: shared lifestyle exposures, treatment-related carcinogenesis, field cancerization, and hereditary predisposition. Together, these converge to elevate SPM risk in each survivor; Figure 1 summarizes this framework schematically. In practice, an individual survivor’s overall risk typically reflects contributions from more than one of these pathways simultaneously (for example, a survivor of tobacco- and alcohol-related esophageal cancer who also received radiotherapy is subject to both field cancerization and treatment-related risk), which is a key reason single-pathway risk estimates should not be summed naively.

### 3.8. Current Forecasting and Individualized Prediction Models

Having mapped where SPM risk concentrates across GI cancer subtypes, we turn now to a harder question: can existing statistical tools predict this risk for an individual survivor? Over the past decade, the Fine and Gray sub-distribution hazard model has become the standard approach for SPM prediction research. It correctly treats death from other causes as a competing event rather than simple censoring, which would otherwise inflate cumulative incidence estimates in populations with substantial non-SPM mortality [26,40]. This framework has produced validated risk-prediction nomograms in several non-GI cancer survivor populations. Breast, prostate, ovarian, gynecologic, and non-small-cell lung cancer survivors each now have published, internally validated tools predicting individualized 3, 5, and 10-year SPM probability from variables such as age, stage, tumor grade, and treatment modality, several with reported concordance indices in the 0.65–0.75 range and decision-curve analyses supporting their net clinical benefit over uniform screening [41,42,43,44]. Within GI oncology specifically, the HCC-focused nomogram described above achieved a concordance index of 0.677 with excellent calibration, making it one of the only such tools built for a GI cancer population to date [26]. We regard a concordance index in this range as informative but modest: it separates higher- from lower-risk patients better than chance, but it leaves considerable individual-level uncertainty that a clinician should not overstate to a patient.

Important limitations persist across this literature, and in our view, several outweigh the field’s recent methodological gains. First, most published nomograms are single-cancer models trained and internally validated on the same registry, typically SEER, with limited external validation across health systems or non-US populations, a real constraint given how much surveillance intensity and background cancer incidence vary internationally. Second, few models incorporate germline or hereditary-risk status. This is a notable omission given how much Lynch syndrome and FAP contribute to metachronous GI malignancy: a nomogram built only from registry-level demographic and treatment variables will systematically under-predict risk in unrecognized syndromic carriers. Third, existing tools remain largely static, single-time-point calculators rather than true multistate models that update as a survivor ages or undergoes further treatment. More flexible recurrent-event and Bayesian semiparametric frameworks that jointly model multiple primaries have been proposed, but they remain confined to specialized hereditary-cancer research cohorts rather than general survivorship populations [45]. Finally, latency-dependent hazard structures are inconsistently modeled. SPM risk for a given site rises and falls at different rates depending on the index cancer and treatment received, and this variation is rarely captured. The latency-trend analyses developed for second primary thyroid cancer are a promising exception that, in our assessment, deserves wider adoption across other GI-relevant second-cancer sites [19,46].

## 4. Discussion

### 4.1. Clinical Implications

Several practical implications for clinicians follow from the evidence summarized above, while bearing in mind that none of these suggestions substitutes for society-specific surveillance guidelines. Gastric, colorectal, and esophageal cancer survivors who received radiotherapy warrant a lower threshold for thyroid examination during routine survivorship visits, given the consistently elevated site-specific thyroid risk described in Section 3.3 and Section 3.4, and 3.6. Colorectal cancer survivors diagnosed before age 50, particularly those with right-sided or hereditary-appearing disease, may warrant closer attention to small-bowel and extracolonic symptoms, given their disproportionately elevated SPM risk relative to older-onset survivors. Universal mismatch-repair or microsatellite-instability testing at the time of colorectal or endometrial cancer diagnosis is among the most effective strategies for identifying Lynch syndrome carriers who require intensified, syndrome-specific surveillance rather than standard post-treatment follow-up. Survivors of esophageal squamous cell carcinoma or head and neck squamous cell carcinoma with heavy combined tobacco and alcohol exposure represent a high-yield population for endoscopic screening of the reciprocal site, particularly where Lugol chromoendoscopy or narrow-band imaging is available. Finally, where a validated nomogram exists for a given cancer type, its output should be interpreted with a clear understanding of its performance characteristics. A concordance index in the 0.65 to 0.70 range, such as the 0.677 reported for the HCC model [26], indicates that, for a randomly selected pair of patients in which one develops an SPM and the other does not, the model assigns the higher predicted risk to the correct patient only about two-thirds of the time [47]. This represents modest discrimination. Such nomograms should therefore be regarded as population-calibrated decision-support instruments rather than definitive predictors of individual patient outcomes. They may help stratify survivors into broad risk categories, inform discussions about surveillance intensity, and identify patients who may warrant further evaluation, such as germline testing, but a predicted probability should not be presented to an individual patient as a forecast of whether a second cancer will develop. In addition, predicted probabilities are only as reliable as the model’s calibration in the population to which it is applied; a nomogram calibrated to SEER may over- or underestimate absolute risk in health systems with different background incidence, surveillance intensity, or case mix, and in patients whose hereditary-risk status has not been established [47].

### 4.2. Future Research Directions

Taken together, the evidence reviewed here supports several concrete priorities for survivorship-focused research in GI oncology. Harmonizing SPM case definitions is the most immediately actionable of these: whether through universal adoption of a standardized latency threshold, or simply routine reporting of results under multiple definitional frameworks, this step alone would substantially improve comparability across registry- and cohort-based studies. The dual-reporting approach outlined in Section 3.1 provides a practical starting point. Risk modeling also needs to catch up. Multistate and competing-risk approaches, already well established in breast, prostate, and gynecologic oncology, should be extended more broadly to colorectal, gastric, esophageal, hepatocellular, and pancreatic cancer survivor cohorts, with external validation across multiple registries and, ideally, non-US populations.

**Figure 2:**
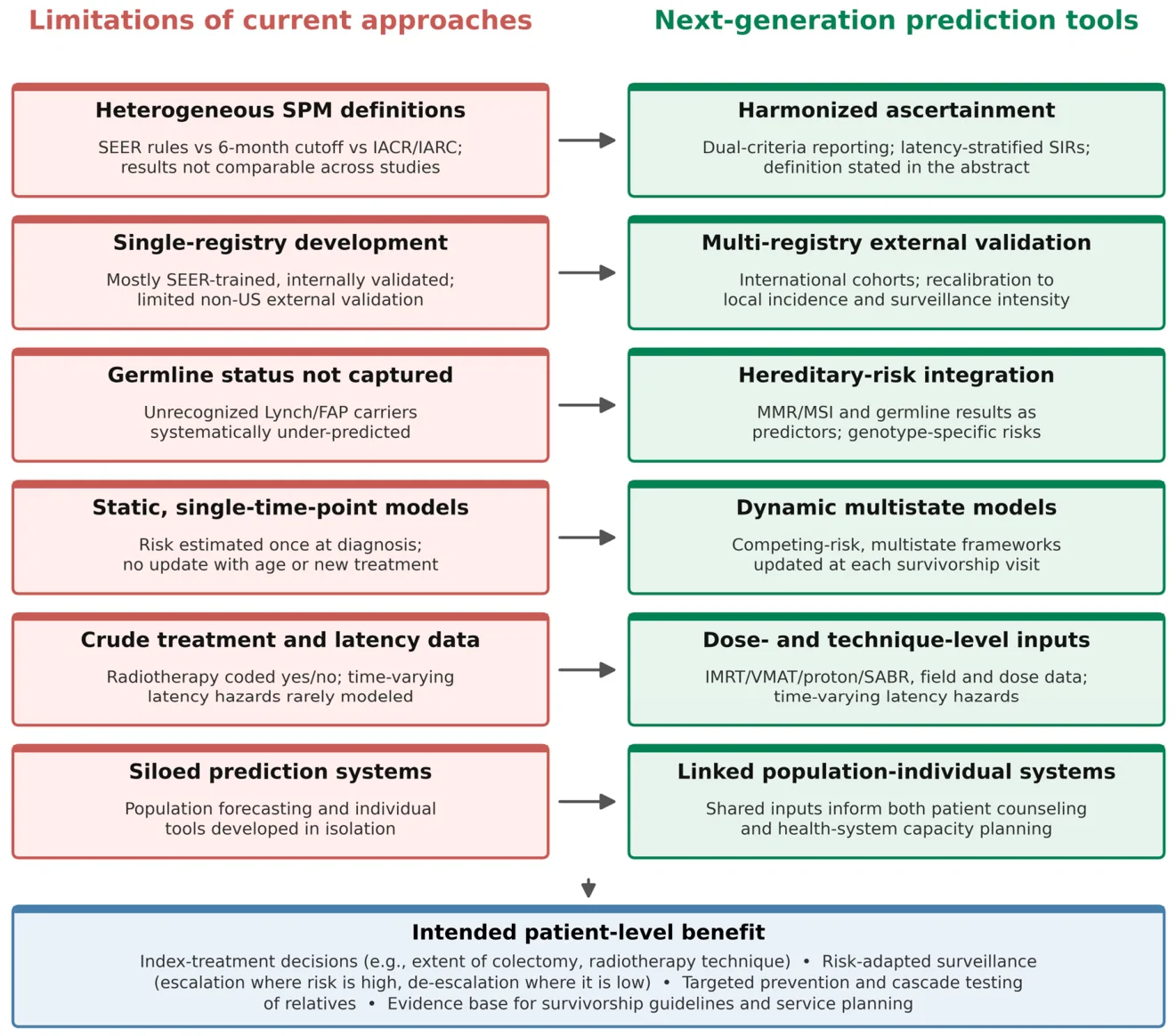
From current limitations to next-generation prediction tools for second primary malignancy (SPM) in gastrointestinal cancer survivors. The left column (red) lists six limitations of current approaches identified in Section 3.1 and Section 3.8: heterogeneous SPM definitions, development and validation within a single registry, absence of germline information, static single-time-point risk estimates, crude treatment and latency data, and separation of population-level forecasting from individual-level prediction. The right column (green) shows the corresponding next-generation approach proposed in Section 4.2. The bottom panel (blue) summarizes the patient-level decisions these tools are intended to inform, as discussed in Section 4.3. FAP, familial adenomatous polyposis; IACR/IARC, International Association of Cancer Registries/International Agency for Research on Cancer; IMRT, intensity-modulated radiotherapy; MMR, mismatch repair; MSI, microsatellite instability; SABR, stereotactic ablative radiotherapy; SEER, Surveillance, Epidemiology, and End Results; SIR, standardized incidence ratio; VMAT, volumetric modulated arc therapy.

Hereditary-risk stratification deserves similar attention. Lynch syndrome, FAP, and other germline findings, where available, should be built into epidemiologic risk estimation and clinical prediction tools rather than treated as a separate surveillance track running in parallel. The genotype-specific pancreatic-risk refinement described in Section 3.7 is a useful model for the kind of granularity the field should be moving toward. Treatment-technique-specific data collection is another gap worth closing, particularly for newer radiotherapy modalities such as proton therapy and stereotactic ablative radiotherapy, whose long-term secondary malignancy profiles remain incompletely characterized. Finally, population-level forecasting and individualized prediction tools continue to develop largely in isolation from one another; linking the two could let health systems anticipate future SPM-related resource needs while still giving clinicians validated, cancer-type-specific surveillance tools. Figure 2 summarizes how each limitation identified in Section 3.8 corresponds to a next-generation approach and how these approaches are intended to translate into patient-level benefit (Section 4.3).

### 4.3. From Better Prediction to Patient Benefit

An important question is how an individual survivor may benefit from improved SPM definitions, risk modeling, and population-level prediction, given that a second primary cancer, once diagnosed, is treated according to its own site, stage, grade, and molecular profile. We agree that improved prediction does not alter the management of an established SPM. Its value lies upstream, in several decisions that are currently made with limited or only population-average information about SPM risk.

The first concerns treatment of the index cancer. In mismatch-repair gene carriers with a first colon cancer, the cumulative risk of metachronous CRC after segmental resection has been estimated at 16% at 10 years, 41% at 20 years, and 62% at 30 years, whereas no metachronous CRC was observed among 50 carriers who underwent extensive colectomy; each additional 10 cm of colon resected was associated with a 31% reduction in risk [39]. Knowing this risk before surgery can inform the choice between segmental and more extensive colectomy. Similarly, for younger patients and known carriers, long-term SPM risk may inform shared decision-making regarding radiation technique, treatment field, or non-radiation alternatives when oncologically appropriate (Section 3.6).

The second concerns the intensity and focus of surveillance. Routine follow-up after GI cancer is designed to detect recurrence and is largely confined to the organ and time window of the index tumor, whereas SPMs arise at other sites and often after routine follow-up has ended. Risk stratification allows surveillance to be extended to sites outside the index field, such as the esophagus in survivors of head and neck squamous cell carcinoma or the thyroid in irradiated survivors; in the esophageal setting, screening has been shown to detect second primary tumors at earlier, more treatable stages [23,24]. Equally, it allows surveillance to be de-escalated where risk is low, as illustrated by the Dutch Lynch syndrome data questioning routine pancreatic surveillance in most carriers [29]. Given that survival after an SPM is poor, with a median survival of less than 4 years after the second diagnosis among CRC survivors [9], earlier detection is where most of the achievable benefit lies.

The third concerns prevention, which extends beyond the individual patient. In Lynch syndrome, aspirin 600 mg daily for 2 to 4 years reduced colorectal cancer incidence over 10 years of follow-up in a randomized trial (intention-to-treat hazard ratio 0.65; per-protocol hazard ratio 0.56) [48]. Chemoprevention, smoking and alcohol cessation, and weight management are most efficient when directed toward survivors at highest risk, and identification of a germline syndrome in a survivor enables cascade testing and surveillance of at-risk relatives. At the health-system level, forecasting the number of survivors expected to develop an SPM can inform endoscopy capacity, survivorship clinic staffing, and guideline development, which will become increasingly important as the number of cancer survivors in the United States is projected to exceed 22 million by 2035 [1].

Direct evidence that risk-adapted SPM surveillance improves overall survival is, however, currently limited to specific high-risk settings, such as Lynch syndrome and esophageal screening in patients with head and neck cancer. Prospective studies comparing risk-adapted with standard surveillance, using endpoints such as stage at SPM diagnosis, treatment burden, and survival, are needed before broader recommendations can be made. Harmonized definitions and well-calibrated risk models are prerequisites for such studies, which is why we regard them as near-term priorities.

### 4.4. Limitations

This review has limitations inherent to its narrative design, as detailed in Section 2: it is not a systematic review with a pre-registered protocol, independent dual-reviewer study selection, or formal quality appraisal of included studies; search terms were applied iteratively rather than through a single reproducible strategy; and publication bias toward statistically significant SIR findings may inflate the apparent strength of some site-specific associations. Because estimates were drawn from studies that differ in registry, follow-up duration, and SPM definition (Table 1), the numeric findings summarized in Table 2 should be read as site-specific reference points rather than directly comparable, pooled figures. Nonetheless, by synthesizing evidence across GI cancer subtypes and across the epidemiologic, genetic, and biostatistical modeling studies, this review aims to provide a foundation for more integrated, survivorship-focused prediction research in this population.

## 5. Conclusions

GI cancer survivors represent a distinct, high-risk population with clinically important, site-specific risk of second primary malignancy that is shaped by shared lifestyle exposures, treatment-related carcinogenesis, field cancerization, and, in a clinically important minority, hereditary predisposition. Current cancer forecasting and individualized prediction frameworks have only begun to incorporate these survivor-specific dynamics, and GI cancer survivors remain underserved relative to other cancer populations with more mature competing-risk modeling tools. Harmonizing SPM definitions, broadening the use of multistate and competing-risk modeling, integrating hereditary-risk stratification, and linking individual- and population-level prediction systems represent achievable near-term priorities. Their value to patients lies not in altering the treatment of an established second cancer, but in informing decisions made before it develops: the extent of index surgery and choice of radiotherapy, the sites and duration of surveillance beyond routine recurrence follow-up, the targeting of preventive measures, and the identification of relatives who share the same hereditary risk. Progress in these areas could enable more precise, risk-adapted surveillance and ultimately improve long-term outcomes for this growing survivor population.

## Figures and Tables

**Table 1:** Comparison of major frameworks used to define and classify second primary malignancies.

Framework	Latency/Synchronous-Metachronous Threshold	Classification Basis	Key Strength	Key Limitation
SEER Multiple Primary and Histology Coding Rules	Site and histology specific coding rules; no single fixed interval applied uniformly	Topography, histology, and laterality-based algorithm	Large, standardized, population-based registry data with long follow-up	Complex rules can under- or over-count borderline second primaries
Cohort-study convention	Fixed 6-month cutoff (synchronous < 6 month; metachronous ≥ 6 month)	Investigator-defined per study protocol	Simple, transparent, and widely used, easing replication	Cutoff is arbitrary and applied inconsistently across studies
IACR/IARC criteria	Topography/histology group-specific rules, conceptually like SEER but not identical	Rule-based, with IARC-specific tumor groupings	Intended to standardize registry reporting internationally	Not universally adopted; classifies some tumor pairs differently than SEER

**Table 2:** Second primary malignancy risk by index gastrointestinal cancer site or hereditary syndrome.

GI Primary Site/Syndrome	Approximate SPM Risk	Most Common Second-Site Cancers	Key Modifying Factors	Ref.
Colorectal (CRC)	~10% cumulative at 10 year; pooled SIR ~1.15	Colorectum, corpus uteri, small intestine (most consistent pooled evidence)	Age < 50, tumor sidedness, radiotherapy	[9,11,14]
Gastric	O/E 1.5–2.0 for thyroid and pancreas; site-specific O/E up to ~5 for stomach	Thyroid, esophagus, small intestine, pancreas	Radiotherapy amplifies esophageal, thyroid, and pancreatic risk	[17]
Esophageal (ESCC)	Markedly elevated SIR for upper aerodigestive tract tumors	Head and neck, lung (reciprocal relationship with HNSCC)	Tobacco/alcohol co-exposure; attenuated in HPV-associated disease	[2,22,25]
Hepatocellular (HCC)	3.95% cumulative SPM incidence (SEER)	Oral cavity/pharynx, stomach, biliary tract, lung, thyroid	Younger age, smaller tumor, earlier stage, surgery (reflects longer survivorship window)	[26]
Pancreatic	<5% at 15 year	Data limited by short survivorship window	Poor baseline prognosis limits SPM ascertainment	[4]
Lynch syndrome (any GI site)	~16% second CRC at 10 year; PDAC/ampullary/distal CC lifetime risk 0–3.4%	Second CRC, endometrial, gastric, urinary tract, small bowel	Genotype-specific risk (e.g., MLH1 vs. MSH6)	[27,28,29]
Familial adenomatous polyposis	Near-100% lifetime CRC risk without colectomy	Duodenal/ampullary adenocarcinoma, desmoid tumors	Timing of prophylactic colectomy	[30,31]

## Data Availability

No new data were created or analyzed in this study. Data sharing is not applicable to this article, as all findings are derived from previously published sources cited in the References section.

## Abbreviations

The following abbreviations are used in this manuscript:

APC	Adenomatous polyposis coli (gene)
CC	Cholangiocarcinoma
CRC	Colorectal cancer
EAR	Excess absolute risk
EPCAM	Epithelial cell adhesion molecule (gene)
ESCC	Esophageal squamous cell carcinoma
FAP	Familial adenomatous polyposis
GI	Gastrointestinal
HCC	Hepatocellular carcinoma
HNSCC	Head and neck squamous cell carcinoma
HPV	Human papillomavirus
IACR	International Association of Cancer Registries
IARC	International Agency for Research on Cancer
IMRT	Intensity-modulated radiotherapy
MLH1, MSH2, MSH6, PMS2	DNA mismatch repair genes
MSI	Microsatellite instability
O/E	Observed-to-expected ratio
PDAC	Pancreatic ductal adenocarcinoma
SABR	Stereotactic ablative radiotherapy
SEER	Surveillance, Epidemiology, and End Results
SIR	Standardized incidence ratio
SPM	Second primary malignancy
VMAT	Volumetric modulated arc therapy
